# Three-Dimensional Bioprinted Controlled Release Scaffold Containing Mesenchymal Stem/Stromal Lyosecretome for Bone Regeneration: Sterile Manufacturing and In Vitro Biological Efficacy

**DOI:** 10.3390/biomedicines10051063

**Published:** 2022-05-03

**Authors:** Elia Bari, Franca Scocozza, Sara Perteghella, Lorena Segale, Marzio Sorlini, Ferdinando Auricchio, Michele Conti, Maria Luisa Torre

**Affiliations:** 1 Department of Pharmaceutical Sciences, University of Piemonte Orientale, Largo Donegani 2/3, 28100 Novara, Italy; elia.bari@uniupo.it; 2 Department of Civil Engineering and Architecture, University of Pavia, Via Ferrata 3, 27100 Pavia, Italy; franca.scocozza01@universitadipavia.it (F.S.); auricchio@unipv.it (F.A.); michele.conti@unipv.it (M.C.); 3 P4P S.r.l., Via Scapolla 12, 27100 Pavia, Italy; 4 Department of Drug Sciences, University of Pavia, Viale Taramelli 12, 27100 Pavia, Italy; sara.perteghella@unipv.it (S.P.); marina.torre@unipv.it (M.L.T.); 5 PharmaExceed S.r.l., Piazza Castello 19, 27100 Pavia, Italy; marzio.sorlini@supsi.ch; 6 Department of Innovative Technologies, University of Applied Sciences and Arts of Southern Switzerland, SUPSI, Lugano University Centre, Campus Est, Via la Santa 1, CH-6962 Viganello, Switzerland

**Keywords:** mesenchymal stem cells, MSC secretome, MSC extracellular vesicles, Lyosecretome, 3D coprinting, poly(ε-caprolactone), alginate hydrogel, bone tissue engineering, bone regenerative medicine

## Abstract

Recently, 3D-printed scaffolds for the controlled release of mesenchymal stem cell (MSC) freeze-dried secretome (Lyosecretome) have been proposed to enhance scaffold osteoinduction and osteoconduction; coprinting of poly(ε-caprolactone) (PCL) with alginate hydrogels allows adequate mechanical strength to be combined with the modulable kinetics of the active principle release. This study represents the feasibility study for the sterile production of coprinted scaffolds and the proof of concept for their in vitro biological efficacy. Sterile scaffolds were obtained, and Lyosecretome enhanced their colonization by MSCs, sustaining differentiation towards the bone line in an osteogenic medium. Indeed, after 14 days, the amount of mineralized matrix detected by alizarin red was significantly higher for the Lyosecretome scaffolds. The amount of osteocalcin, a specific bone matrix protein, was significantly higher at all the times considered (14 and 28 days) for the Lyosecretome scaffolds. Confocal microscopy further confirmed such results, demonstrating improved osteogenesis with the Lyosecretome scaffolds after 14 and 28 days. Overall, these results prove the role of MSC secretome, coprinted in PCL/alginate scaffolds, in inducing bone regeneration; sterile scaffolds containing MSC secretome are now available for in vivo pre-clinical tests of bone regeneration.

## 1. Introduction

Three-dimensional bioprinting is a state-of-the-art additive manufacturing technique that allows the creation of three-dimensional (3D) physical objects by depositing biomaterials in a layer-by-layer fashion [1,2,3,4]. As the constructs created by 3D bioprinting have characteristics (e.g., more highly-controlled microstructures) closer to natural tissues or organs, this technique is increasingly becoming an important preferred tool in individualized fabrication [5,6,7]. In this regard, bone tissue engineering (TE) is an emerging 3D (bio)printing application field that focuses on the manufacturing of implantable scaffolds to recapitulate bone defects due to trauma or disease [8,9,10,11]. From a biological point of view, the printed scaffold should promote in vitro proliferation and differentiation when cells are seeded on the scaffold and the in vivo colonization when implanted. Therefore, to satisfy these requirements, the scaffold must be biocompatible and biodegradable [12,13,14]; in addition, it should promote osteogenesis, osteoinduction (supporting stem cells to differentiate into osteoblasts), and osteoconduction (supporting the ingrowth of capillaries and cells to form bone) [15,16,17].

Many factors could influence the scaffold’s biological properties, including biomaterial(s) selection, scaffold design (e.g., porosity, pore size), and fabrication methods. A wide range of biocompatible and biodegradable synthetic thermoplastic polymers are currently available for 3D printing scaffolds for bone TE applications. Among these, poly(ε-caprolactone) (PCL) is one of the most commonly used in this field due to its ease of handling, biocompatibility, stability, hydrolysis, and enzymatic digestion similar to the bone healing range [18,19]. In addition, it is approved by US Food and Drug Administration (FDA) [20]. Recently, additive manufacturing technology (e.g., 3D printing and bioprinting) has been employed to manufacture scaffolds with complex geometry and internal interconnected porous structures [21,22]. The pore size is crucial for proper cell adhesion, proliferation, differentiation, and scaffold colonization. In fact, according to the literature, the scaffold filament distance could promote cell proliferation, migration, and nutrient transport ranging between 100 and 500 mm [23].

In this context, one of the outstanding problems with both in vitro culture and in vivo grafting of the printed scaffold is that it does not always end successfully due to the inability of cells to properly colonize the scaffold, resulting in tissue necrosis [24,25]. To overcome this issue, in previous studies [26], we proposed enriching scaffolds with the MSC secretome, as it contains growth factors, cytokines, other proteins, and oligonucleotides (as free soluble factors and/or loaded in extracellular vesicles, EVs) that can promote cell proliferation and differentiation, and thus sustain scaffold colonization and tissue regeneration. In detail, we 3D-coprinted a scaffold made by PCL and alginate-based hydrogel containing Lyosecretome—a freeze-dried formulation of MSC secretome. Alginate was chosen due to its ability to form crosslinked hydrogels when hydrated and in the presence of divalent cations [27] that allow the controlled release of MSC secretome.

In this context, another issue in this field is the fabrication of scaffolds in sterile conditions: if a thermostable scaffold (e.g., titanium scaffold) can be sterilized in the final container, this cannot be for scaffold made with the biomaterials previously mentioned as they are sensitive to high temperatures. For this reason, many products that theoretically could have been used in TE, in practice, could not be used to carry out in vitro cell tests and, much less, in vivo or clinical trials. Thus, very profitable products have never achieved a clinical application due to the difficulties in producing sterile scaffolds.

In this work, the feasibility study of the coprinting process under sterile conditions was performed. Then, the in vitro efficacy of coprinted PCL/alginate scaffolds enriched with Lyosecretome was investigated by MSC osteogenic differentiation and matrix production tests.

## 2. Materials and Methods

### 2.1. Materials 

PCL pellets (50 kDa) were purchased from Cellink AB (Gothenburg, Sweden). The antibiotics, culture media, and trypsin-EDTA used for cell culture were purchased from Euroclone (Milan, Italy). Human platelet lysate (PL) was purchased from Carlo Erba reagents (Milan, Italy). Mannitol, Nile red, sodium alginate, calcium chloride, and protamine were from Sigma Aldrich (Milan, Italy). 

### 2.2. Lyosecretome Preparation and Characterization

#### 2.2.1. MSC Expansion and Secretome Collection

Lyosecretome was prepared from adipose-derived MSCs (AD-MSCs). The adipose tissue was collected from abdominoplasty patients who had previously signed informed consent (ASST Grande Ospedale Metropolitano Niguarda, Milan, Ref. 12 November 2009). MSCs were isolated from adipose tissue according to previously reported procedures [8], seeded into flasks (10,000 cells/cm^2^), and cultured at 37 °C and 5% CO_2_ in DMEM/F12 minimal medium plus 5% *v*/*v* PL and antibiotics (1% *v*/*v* penicillin/streptomycin and 1% *v*/*v* amphotericin B) until passage 3. Secretome release was induced by culturing MSCs in a culture medium without platelet lysate for 48 h. The conditioned media were collected and pooled, then MSCs were detached with trypsin-EDTA and tested to evaluate cell viability, sterility (according to Eu. Ph. 9.0, 2.6.27), and concordance with the International Society for Cellular Therapy [28] criteria. 

#### 2.2.2. MSC Secretome Ultrafiltration and Lyophilization

After centrifugation at 3500× *g* for 10 min, conditioned media were ultrafiltered by tangential flow filtration (KrosFlo^®^ Research 2i system, Spectrum Laboratories, Milan, Italy) using a filtration module with a molecular weight cut-off (MWCO) of 5 kDa (Spectrum Laboratories, Milan, Italy). When a concentration of 0.5 × 10^6^ cell equivalents per mL was reached, the concentration step was stopped, and the samples were then diafiltered using sterilized ultrapure water. Finally, cryoprotectant (0.5% *w*/*v* mannitol) was added to the concentrated and purified secretome, which was then frozen at −80 °C and freeze-dried (Christ Epsilon 2-16D LSCplus) at 8 × 10^−1^ mbar and −50 °C for 72 h. Each mg of freeze-dried powder corresponds to 0.1 × 10^6^ cell equivalents (calculated as the ratio between the total cell number used and the obtained milligrams of Lyosecretome).

#### 2.2.3. Lyosecretome Characterization

Lyosecretome protein and lipid content was assessed using the BCA Protein Assay Kit from Thermo Fisher Scientific (Milan, Italy) and the Nile Red assay, respectively. Both methods have been validated previously [26]. Analyses were performed in triplicate.

Nanoparticle tracking analysis (NTA, NanoSight NS 300 equipment, Malvern Panalytical Ltd., Malvern, UK) was used to determine the EV concentration and particle size of the Lyosecretome after dispersion in water at 1 mg/mL. Analyses were conducted in triplicate at room temperature.

#### 2.2.4. Lyosecretome Microbiological Controls

According to European Pharmacopoeia (EuPh 2.6.2), a microbiology test was conducted on Lyosecretome; bacterial endotoxins were measured using the Limulus Amebocyte Lysate (LAL) test (EuPh 2.6.14) to assure apyrogenicity. In addition, the NAT test detected possible mycoplasma contamination according to the provisions of European Pharmacopoeia (EuPh 2.6.7).

### 2.3. Scaffolds Design and Fabrication 

**Figure 1:**
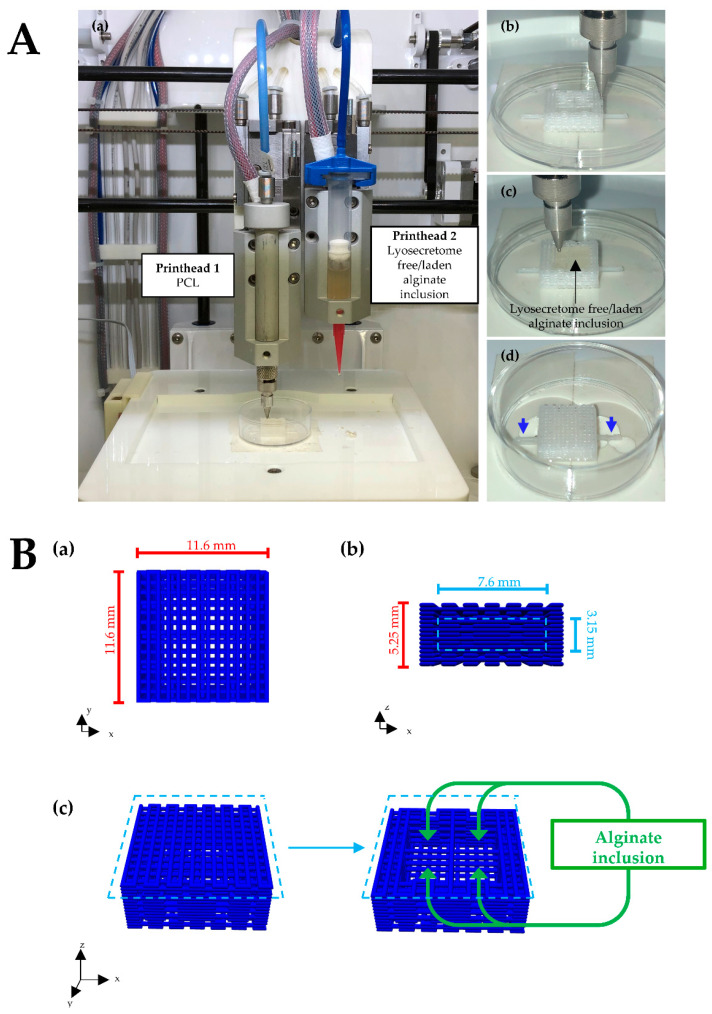
(**A**) Three-dimensional coprinting setup and process of PCL and alginate scaffold. (**a**) PCL and Lyosecretome-laden/free alginate were 3D printed using the PH1 and PH2, respectively. (**b**) The first part of the PCL scaffold was printed, forming the four wells in which alginate will be extruded. (**c**) After alginate extrusion, the last part of the scaffold was printed to cover the wells and create the inclusion. (**d**) Printed scaffold ready to be crosslinked with calcium chloride and protamine solutions. Blue arrows indicate two linear protrusions printed to block the scaffold into the well plate, avoiding floating during in vitro culture. (**B**) Scaffold geometry and dimensions: (**a**) planar, (**b**) sectional, and (**c**) total view of the scaffold with a focus on wells in which alginate will be extruded to form the inclusions.

Porous parallelepiped-shaped PCL scaffolds (11.6 mm × 11.6 mm × 5.25 mm) with alginate/Lyosecretome inclusion were 3D-coprinted following the process described in our previous study [26]. Briefly, an extrusion-based 3D bioprinter (Cellink INKREDIBLE+) equipped with dual heated printheads (PHs) was used. The first PH was filled with PCL pellets and heated at 90 °C. The second PH was filled with Lyosecretome-laden alginate prepared by dissolving 1.25 mg of Lyosecretome for each ml of a 10% *w*/*v* alginate solution (Figure 1A). PCL was printed into a parallelepiped-shaped structure of 15 layers of 0.35 mm in height, and the distance of the fiber was fixed at 0.4 mm. The following printing parameters were used: extrusion pressure of 350 kPa; conical nozzle diameter of 0.5 mm; printing speed of 45 mm/min; and printing temperature of 90 °C. The Lyosecretome-laden alginate was printed into one internal well of 7.6 mm by diameter and 5.4 mm by height using the following printing parameters: extrusion pressure of 20 kPa; conical nozzle diameter of 0.41 mm; and printing at room temperature. After printing, the scaffold was double crosslinked with 2% *w*/*v* CaCl_2_ and subsequently with a 5% *w*/*v* protamine solution.

Although the 3D bioprinter is equipped with a UV light (365 nm), HEPA filter, and positive air pressure to ensure a sterile environment during the printing process, all the procedures were conducted under a laminar conditions flow hood in a B cleanroom suite. Materials with a proved sterility validation document were employed or sterilized before use. In particular, the PCL was sterilized following a process implemented in a previous study [29], and sodium alginate was pasteurized. In addition, CaCl_2_ and protamine solutions were filtrated. Three different batches were produced to simulate process validation, although performed at a laboratory small-scale.

### 2.4. Scaffold Microbiological Controls

Before performing the biological assessment, the sterility of the printed scaffolds was evaluated following the United States Pharmacopeia (USP) [30] method described in our previous study [29]. Briefly, 3D-printed scaffolds with Lyosecretome-laden alginate inclusion printed under sterile conditions were compared with scaffolds printed under no sterile conditions. Each scaffold (*n* = 3 per group) was placed in a sterile tube, immersed in 2 mL of soybean-casein digest medium (SCDM), and incubated for 14 days at 25–27 °C and 37 °C. Scaffolds were monitored every two days for any evidence of the growth of microorganisms. Two tubes filled with SCDM only (no scaffold inside) were used as a control. Following the EuPh 2.6.27 and 2.6.1 chapters, the scaffolds were tested for sterility and microbiological tests. The Limulus amebocyte lysate test (EuPh 2.6.14) measured the bacterial endotoxins, while the NAT test was used to detect mycoplasma contamination (EuPh 2.6.7).

### 2.5. Biological Assessment

The biological assessment was performed to evaluate the influence of Lyosecretome on the osteogenic differentiation of MSCs. To this end, we 3D-coprinted PCL scaffolds with Lyosecretome-free and -laden alginate inclusion. The scaffold with the inclusion of Lyosecretome-free-laden alginate is referred to as the control (CTR). 

#### 2.5.1. Seeding of AD-MSCs

The scaffolds were placed inside a 24-well plate, and 90,000 MSCs were seeded onto their upper surface. The cellular suspension was allowed to be absorbed by the porous alginate substrates for 2 h in a humidified atmosphere with 5% CO_2_ at 37 °C. Then, complete culture media was added to each well. Even if preliminary investigations revealed that above 95% of seeded MSCs attached to the scaffold, to avoid residual cells on the plastic surface below, 24 h after seeding, scaffolds were moved from the original wells to new wells.

#### 2.5.2. Evaluation of cell Adhesion by Scanning Electron Microscopy (SEM)

After 24 h, the cell/scaffold constructs were prepared for morphological investigation by SEM. Briefly, each scaffold was washed with PBS, fixed for 3 h with 3% *v*/*v* glutaraldehyde at 4 °C, and dehydrated with graded ethanol series (50, 70, 90, and 100% *v*/*v*). After being coated with chromium using a high-vacuum Quorum Q150T ES Plus sputtering system, the samples were observed by SEM MIRA3 (Tescan, Brno, Czech Republic) operating with an acceleration voltage of 5 kV and an EDS detector (X-max 50 mm^2^, Oxford Instruments, Oxford, UK). Three independent experiments were performed for each condition (Lyosecretome and CTRL).

#### 2.5.3. Osteogenic Differentiation

An osteogenic medium was prepared with 2% *v*/*v* FBS, dexamethasone (5 nM), ascorbic acid (2.5 µg/mL), and β-glycerophosphate (0.5 mM). It was added to the cell/scaffold constructs and then incubated in a humidified atmosphere with 5% CO_2_ at 37 °C. 

##### Alizarin Red Staining

The alizarin red staining was performed 14 and 28 days after osteogenic differentiation to reveal the deposition of calcium-rich mineralized matrix. The samples were washed with PBS, fixed with 70% *v*/*v* ethanol for 60 min at room temperature, and stained with alizarin red 40 mM for 10 min at pH 4–4.2. Next, the alizarin red in excess was removed by rinsing with distilled water and PBS. Finally, the alizarin red bound to the mineral matrix was dissolved by treating samples with 10% *w*/*v* cetylpyridinium chloride at room temperature for 15 min; a third-degree polynomial equation with R^2^ = 0.99 was built from a concentration vs. absorbance plot obtained from standard alizarin red solutions to extrapolate the alizarin red concentration. Each condition (Lyosecretome and CTRL scaffolds) was tested in three independent experiments.

##### Confocal Microscopy

After 14 and 28 days of osteogenic differentiation, the mineralized matrix deposition was assessed by confocal microscopy. After being washed with PBS and fixed with 3% *v*/*v* glutaraldehyde at 4 °C, each scaffold was treated with the following reagents: TRITC-phalloidin (diluted 1:7000 in PBS containing 0.1% *v*/*v* Triton X-100, 0.1% *w*/*v* BSA, and 10% *v*/*v* FBS), 100 µL of Hoechst 33,258 (diluted 1:10,000 in PBS), and Osteoimage^TM^ mineralization assay according to the manufacturer’s instructions. TRITC-phalloidin was used to stain actin cytoskeleton, Hoechst 33,258 was used to stain cell nuclei, and the Osteoimage^TM^ was used to stain hydroxyapatite. Scaffolds were imaged using a confocal laser scanning microscope (CLSM) (Leica TCS SP2, Leica Microsystems, Wetzlar, Germany). The following parameters were used: λ_ex_ = 540/5 nm and λ_em_ = 570/3 nm for TRITC-phalloidin; λ_ex_ = 346 nm and λ_em_ = 460 nm for Hoechst 33,258; and λ_ex_ = 492 nm and λ_em_ = 520 nm for Osteoimage^TM^. The software (Leica Microsystem, Wetzlar, Germany) associated with the microscope processed the acquired images. Each condition (Lyosecretome and CTRL scaffolds) was tested in three independent experiments.

##### Dosage of Osteocalcin (OCN) by ELISA

The concentration of OCN was determined in cell supernatants of cell/scaffold constructs after 14 and 28 days of osteogenic differentiation. An ELISA kit was used according to the manufacturer’s instructions. Each condition (Lyosecretome and CTRL scaffolds) was tested in three independent experiments.

#### 2.5.4. Statistical Analysis

Raw data were processed by STATGRAPHICS XVII (Statpoint Technologies, Inc., Warrenton, VA, USA) with a general linear analysis of variance model (ANOVA). An LSD (least significant difference) test was coupled to estimate the differences between means. Data regarding the alizarin red assay and OCN were elaborated considering the time and the scaffold type (Lyosecretome or CTRL) as fixed factors with the alizarin red or OCN concentration as the response variable. Statistical significance was considered at *p* < 0.05. Data are reported as mean values ± standard deviation (from at least three independent experiments) unless otherwise specified.

## 3. Results and Discussion

The combination of MSC secretome with 3D-printed scaffolds allows next-generation tissue-engineered implants with an enhanced biological performance to be obtained [26]. In this regard, this work investigated the ability of MSC secretome to support the adhesion, proliferation, and osteogenic differentiation of MSCs seeded on PCL scaffolds. To this end, MSC secretome was first converted into a ready-to-use powder, Lyosecretome, which contained 22.00 ± 3.097 µg of proteins and 3.44 ± 0.0741 µg of lipids per mg of powder (mean values ± SD, *n* = 3). EVs showed a mean diameter of 202.0 ± 1.7 nm, and the d_10_, d_50_, and d_90_ were 110.2 ± 5.3, 166.0 ± 2.8 nm, and 342.0 ± 12.8 nm, respectively. Such results confirm that both proteins and lipids were retained during the ultrafiltration process, and the EV population had the typical size reported in the literature [31]. Lyosecretome microbiological controls indicated that the product was sterile and free of endotoxins and mycoplasma contaminations.

Lyosecretome was added to alginate to prepare the bioink and coprinted with PCL to fabricate scaffolds of a parallelepiped shape. We performed microbiological control to assess the sterility of the printed scaffold before biological characterization following the method established by USP. After 14 days, scaffolds fabricated in non-sterile conditions showed turbidity due to aerobic microbial growth. Meanwhile, the medium containing scaffolds printed in sterile conditions did not show turbidity throughout the incubation period, so no evidence of aerobic microbial growth was found. For scaffolds printed in sterile conditions, the microbiological analyses revealed that all samples were sterile, without mycoplasma contamination, and with a bacterial endotoxin amount lower than 9 EU/mL. 

**Figure 2:**
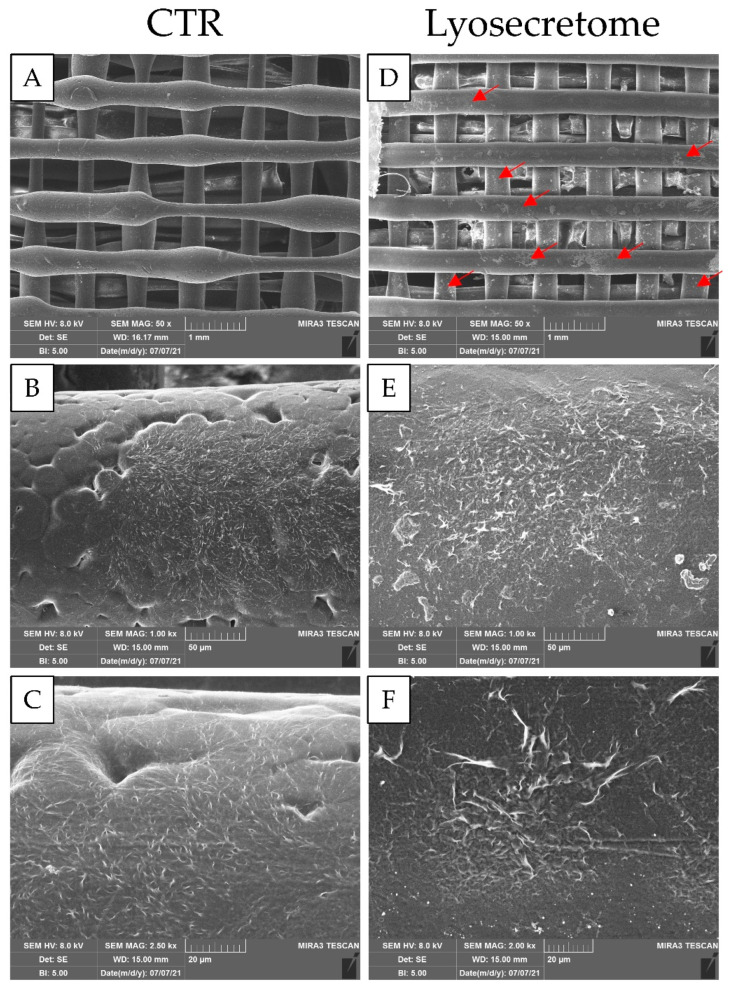
SEM morphological and structural characterizations of Lyosecretome and CTR scaffolds seeded with MSCs and cultured in osteogenic medium for 24 h. Magnifications: 50× (**A**,**D**), 100× (**B**,**E**), 2500× (**C**), and 2000× (**F**). The red arrows indicate the cells that colonized the PCL fibers. Scale bars: 1000 (**A**,**D**), 50 (**B**,**E**), and 20 (**F**) µm.

The osteoinductive properties of the scaffolds were then characterized. At first, the effective seeding of MSCs onto scaffolds (and the effect of Lyosecretome on this) was assessed by SEM. As evidenced in Figure 2, most seeded MSCs were absorbed into the alginate matrix. Consequently, few cells, especially for the CTR scaffolds, are noticeable on the PCL fibers. Conversely, in the Lyosecretome scaffolds, more cells also colonized the PCL fibers (Figure 2D, red arrows). Furthermore, higher magnifications showed that MSCs appeared large and flattened on the PCL surface with a spread morphology; the irregular edges of the cells suggested the formation of filopodia, with more frequent and complex cellular processes, overall indicating the cytocompatibility of the scaffold (Figure 2).

**Figure 3:**
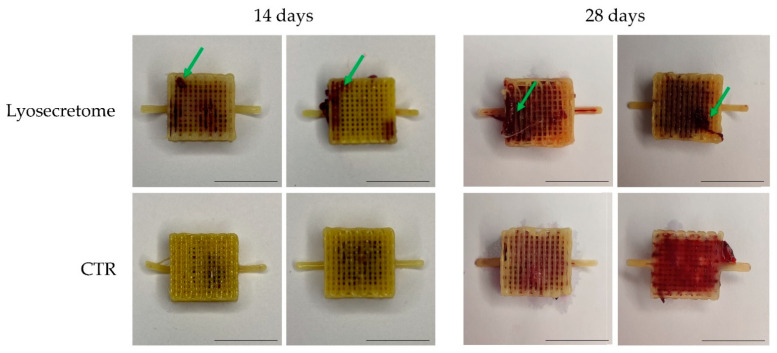
Representative images for the macroscopic evaluation of the mineralized matrix deposition (green arrows) colored by alizarin red on Lyosecretome or CTR scaffolds. Scale bar: 1 cm.

Alizarin red staining demonstrated calcium phosphate deposition by MSCs on the Lyosecretome or CTR scaffolds upon culturing in an osteogenic differentiation medium. Macroscopically, it was evident that the mineralized matrix was highly deposited in the Lyosecretome scaffolds (Figure 3). In detail, after 14 days, CTR scaffolds showed the mineralized matrix only inside the scaffold, where, according to SEM investigation, the majority of MSCs were localized. Conversely, for the Lyosecretome scaffolds, the mineralized matrix was also evident on the PCL fibers. After 28 days, both scaffolds were highly mineralized; however, for the CTR one, the mineralization was mainly in the porosity and was hardly detectable on the surface of the PCL fibers. 

**Figure 4:**
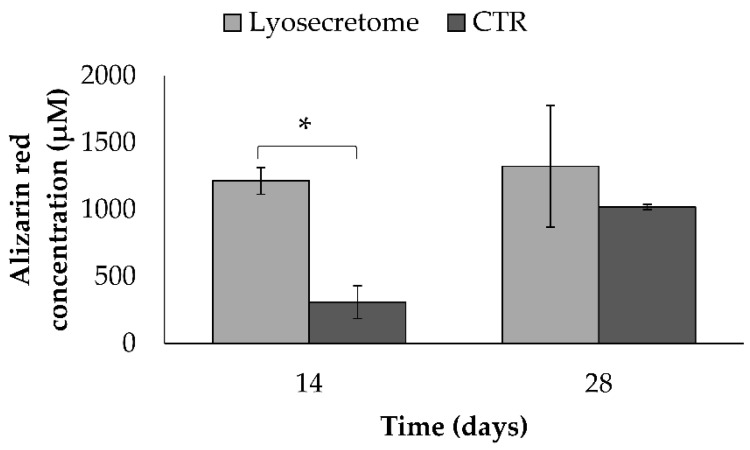
Alizarin red staining for samples cultured in osteogenic differentiation medium with Lyosecretome or without (CTR) for 14 and 28 days. Multifactor ANOVA (time and treatment, mean values ± LSD, *n* = 3) was used. * *p* < 0.001.

Next, the alizarin red bound to the mineralized matrix was dissolved and quantified (Figure 4). According to macroscopic observations, after 14 days, a significantly higher amount of alizarin red was detected in the Lyosecretome scaffolds. At 28 days, no significant differences were revealed between the Lyosecretome and CTR scaffolds. 

**Figure 5:**
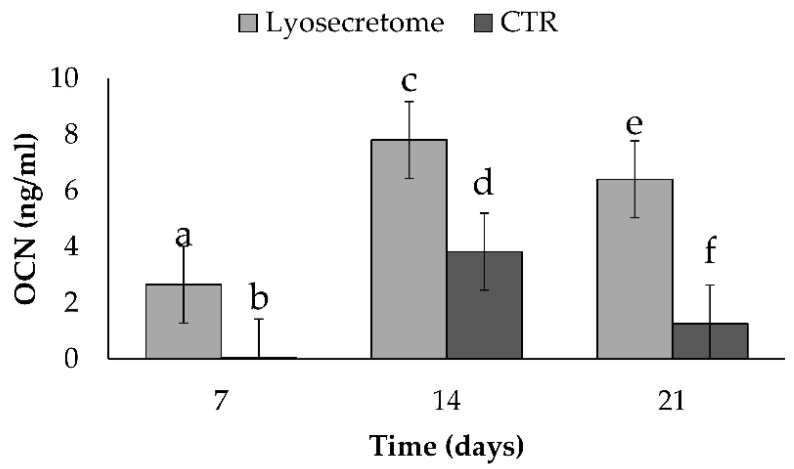
Osteocalcin concentration for Lyosecretome or CTR scaffolds cultured in osteogenic differentiation medium for 7, 14, and 21 days. Multifactor ANOVA (time and treatment, mean values ± LSD, *n* = 3) was used. Different letters (a, b, c, d, e, f) indicate a significant difference between the means (*p* < 0.05), while the same letters indicate no significant difference between the means (*p* > 0.05).

Once MSCs are differentiated into osteoblasts, specific bone matrix proteins are expressed, such as OCN [32,33,34]. The production of OCN increased significantly for both the Lyosecretome and CTR scaffolds until the 14th day and then decreased at 21 days (Figure 5). The amount of OCN produced by MSCs seeded on the Lyosecretome scaffold was significantly higher at all the times considered. The higher expression of OCN and the higher deposition of mineralized matrix observed by alizarin red are in accordance. 

**Figure 6:**
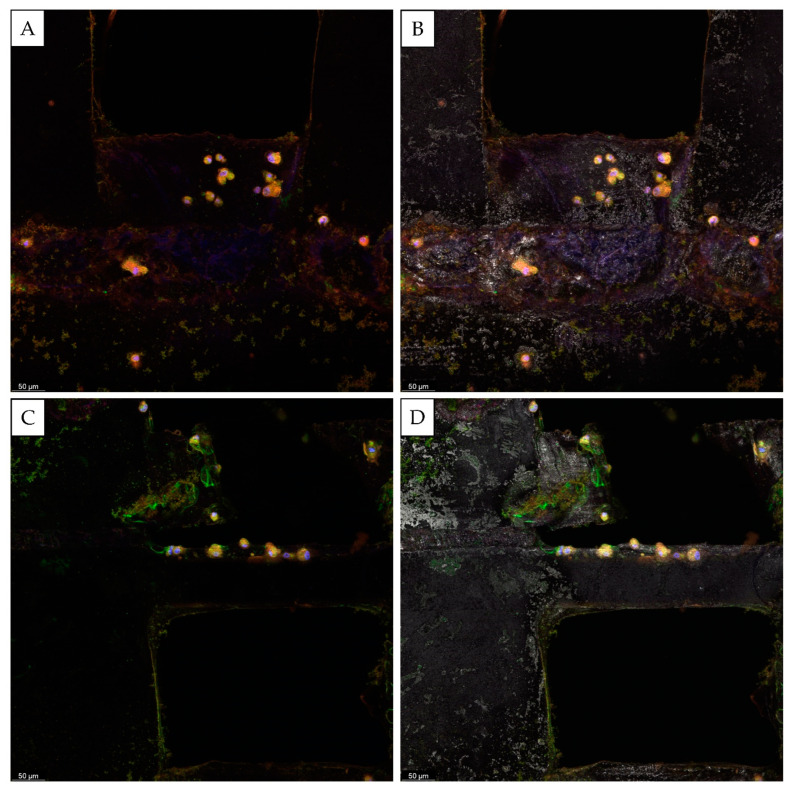
Confocal microscopy images of CTR scaffolds seeded with MSCs and cultured in an osteogenic medium for 28 days. Cell nuclei are stained in blue; the actin cytoskeleton is stained in red; and the mineralized matrix is stained in green (**A**–**D**). In (**B**,**D**), the material of the scaffold is evidenced in grey. Scale bar: 50 µm.

**Figure 7:**
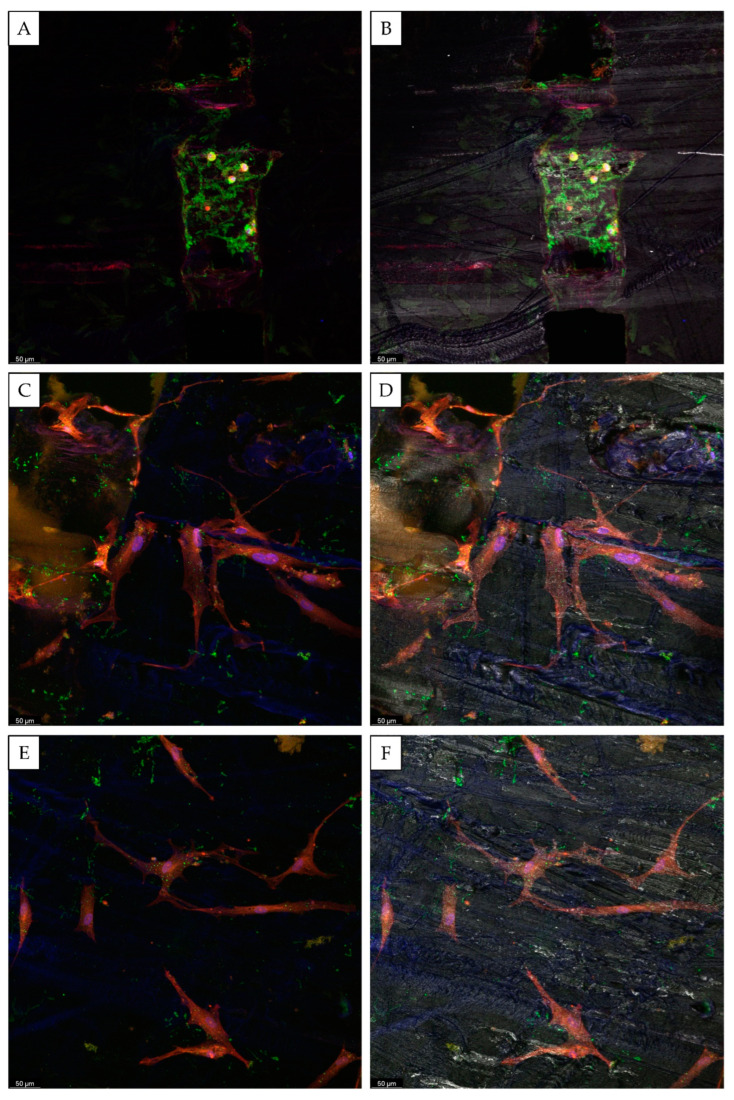
Confocal microscopy images of Lyosecretome scaffolds seeded with MSCs and cultured in an osteogenic medium for 28 days. Cell nuclei are stained in blue; the actin cytoskeleton is stained in red; and the mineralized matrix is stained in green (**A**–**F**). In (**B**,**D**,**F**), the material of the scaffold is evidenced in grey. Scale bar: 50 µm.

Confocal microscopy further confirmed those results (Figure 6 and Figure 7). After 28 days, the presence of the mineralized matrix in the control scaffolds was revealed by Osteoimage^TM^ coloration (in green). The deposition of the mineralized matrix was evident in the proximity of the scaffold’s pores, and it was not detectable on the surface of PCL fibers (Figure 7). For the Lyosecretome scaffold, the amount of mineralized matrix deposited in the proximity of the pores was higher. The mineralized matrix was also detectable on the PCL fibers’ surface (Figure 6). This result is in accordance with the SEM morphological investigation and the macroscopic observation of the mineralized matrix reported in Figure 2 and Figure 3, highlighting cell colonization and hydroxyapatite deposition only in the pores for CTR even onto the PCL fibers’ surface for the Lyosecretome scaffolds. However, it has to be noted that the deposition of the mineralized matrix is prevalent in the pores of the scaffold. This result is in line with previous work, which demonstrated a higher in vitro deposition of hydroxyapatite in the pores of a titanium cage compared to the surface [35]. Also, Li and colleagues demonstrated in vivo that an increase in porosity and pore size enhanced the osteoconductive properties of scaffolds [36]. This is likely the consequence of the increased permeability of nutrients and the higher concentration of the differentiating factors of Lyosecretome, which is laden inside the pores of the scaffold. This may be a limitation, as it is important to achieve proper mineralization across the whole scaffold rather than heavy mineralization in distinct spots. However, the duration of the experiment has to be considered, and, with more time, osteoclasts would probably be able to colonize the entire scaffold and cover it with the mineralized matrix.

It also has to be noted that in this work, a mineralized matrix was produced by osteoblast-differentiated MSCs after 28 days, while in a previous work by us, the mineralization of titanium cages occurred only after 56 days [35]. This observation may suggest that when mechanical resistance is adequate, it is better to choose PCL scaffolds over titanium ones to increase osteoinduction. In this regard, Hung and colleagues compared the effectiveness of titanium mesh and a polycaprolactone-tricalcium phosphate (PCL/TCP) implant for repairing traumatic orbital fractures and revealed no statistically significant difference between the two materials, concluding that both of them can be used to fabricate biodegradable fixators for the reconstruction of orbital wall fractures [37,38]. Conversely, Hyung Shim and colleagues reported that new bone areas around implants were higher for PCL membranes than for titanium mesh membranes [38].

In conclusion, the developed technological process can be completed under sterile conditions, and this is very important for a product intended for implantation. Indeed, ensuring the sterility of such products is not always easy, mainly when the finished product contains thermolabile substances (such as both Lyosecretome and alginate in our case). Also, similar results regarding the ability of MSC secretome to support cell adhesion and osteogenic differentiation have been obtained for titanium cages [35] and a bovine bone-derived matrix (SmartBone^®^) [39]: MSC secretome markedly improved the osteoinductive potency of the scaffolds. Therefore, Lyosecretome has proved to be a valid tool for improving the osteoinduction and osteoconduction properties of medical devices intended for bone regenerative medicine.

## 4. Patents

The content of this work is covered by the Italian patent-pending application number 102021000005441 (filing date 9 March 2021) and the international patent-pending application number PCT/IB2022/052092.

## Data Availability

The data presented in this study are contained within the article.
